# Cost‐effective and simple flow cytometry quantification of receptor‐mediated autophagy using fluorescent tagging

**DOI:** 10.1002/2211-5463.13958

**Published:** 2024-12-23

**Authors:** Mija Marinković, Ana Rožić, Denis Polančec, Ivana Novak

**Affiliations:** ^1^ Faculty of Science University of Split Croatia; ^2^ School of Medicine University of Split Croatia; ^3^ Greyledge Europe Ltd Zagreb Croatia

**Keywords:** BNIP3L/NIX, flow cytometry, fluorescent tagging, receptor‐mediated mitophagy

## Abstract

Mitophagy, a selective clearance of damaged or superfluous mitochondria via autophagy machinery and lysosomal degradation, is an evolutionarily conserved process essential for various physiological functions, including cellular differentiation and immune responses. Defects in mitophagy are implicated in numerous human diseases, such as neurodegenerative disorders, cancer, and metabolic conditions. Despite significant advancements in mitophagy research over recent decades, novel and robust methodologies are necessary to elucidate its molecular mechanisms comprehensively. In this study, we present a detailed protocol for quantitatively assessing mitophagy through flow cytometry using a mitochondria‐targeted fluorescent mitophagy receptor, GFP‐BNIP3L/NIX. This method offers a rapid alternative to conventional microscopy or immunoblotting techniques for analyzing mitophagy activity. Additionally, this approach can theoretically be adapted to utilize any fluorescent‐tagged selective autophagy receptor, enabling the direct and rapid analysis of various types of receptor‐mediated selective autophagy.

## Graphical abstract

Mitophagy, the selective degradation of damaged mitochondria, is crucial for cellular health and function. This study introduces a rapid and efficient flow cytometry method using GFP‐BNIP3L/NIX to accurately quantify mitophagy. Our approach can be adapted to use any fluorescent‐tagged selective autophagy receptor, enabling direct and rapid analysis of various types of receptor‐mediated selective autophagy in diverse biological conditions.

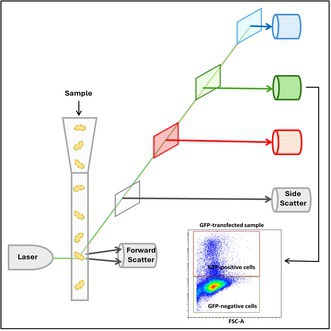

**Abbreviations**
BNIP3L/NIX: BCL2/adenovirus E1B 19‐kDa‐interacting protein 3‐like
CCCP: carbonyl cyanide m‐chlorophenylhydrazone
DMEM: Dulbecco's modified Eagle medium
FBS: fetal bovine serum
FSC‐A: forward scatter area
FSC‐H: forward scatter height
LC3: light chain 3
LIR: LC3‐interacting region
PI: propidium iodide
PINK1: PTEN‐induced putative kinase 1
SSC‐A: side scatter area
SSC‐H: side scatter height
TIMM23: translocase of inner mitochondrial membrane 23
TM: transmembrane domain
TOMM20: translocase of outer mitochondrial membrane 20

As the powerhouses of nearly every human cell, mitochondria are essential intracellular organelles whose pivotal role in maintaining energy homeostasis places them in the center of cell integrity, function, and survival. Because energy provision is the primary requirement for every aspect of cellular function, multiple sophisticated systems for preserving mitochondrial quality control have evolved including, the ubiquitin‐proteasome system, mitochondrial dynamics, and mitophagy. Mitophagy, a specialized form of lysosome‐mediated selective degradation, targets damaged or redundant mitochondria, packaging them into specific autophagosome vesicles for elimination. Despite its pivotal role in mitochondrial homeostasis, mitophagy‐driven elimination of mitochondria takes an important part in many biological processes including early embryonic development, cell differentiation, inflammation, or apoptosis. Therefore, it is not surprising that mitophagy dysfunctions are associated with various pathological conditions ranging from neurodegenerations to cancers [1].

Mitophagy can be induced by different stress stimuli, including hypoxia, nutrient deprivation, high levels of reactive oxygen species, or DNA damage, but can also be a part of programmed mitochondrial removal during differentiation of specialized cell type [2]. In the first scenario, PTEN‐induced putative kinase 1 (PINK1)/Parkin response pathway is activated [3], while during programmed degradation of mitochondria, mitophagy receptors play a central role in mitochondrial removal [4]. Several outer mitochondrial membrane proteins have been identified as mitophagy receptors [5, 6, 7, 8, 9, 10], but most extensively studied mitophagy receptor, BCL2/adenovirus E1B 19‐kDa‐interacting protein 3‐like (BNIP3L/NIX), is crucial for the programmed removal of healthy mitochondria during the terminal differentiation of erythrocytes [11, 12]. Some recent studies have intensively investigated the mechanisms of BNIP3L/NIX regulation [13, 14] as well as its potential roles in cellular physiology and pathology [2, 7, 8, 14, 15].

Significant advancements in the field of mitophagy over the past decade have yielded a deeper understanding of the biochemical mechanisms underlying this process and its implications in disease development. However, the absence of robust and powerful methodologies for studying mitophagy remains a major hurdle in the field. As well as autophagy, mitophagy can similarly be monitored by two different approaches: direct observation of mitophagy structures or quantification of mitophagy‐dependent degradation of mitochondria and mitochondrial proteins. Current methodologies, such as electron and fluorescence microscopy, western blotting, mtDNA quantification, and citrate synthase activity assays, are valuable tools for mitophagy assessment. However, they are not without limitations, including quantification challenges, research bias, and *in vitro* study restrictions [16]. While electron microscopy provides direct visualization of autophagosome‐engulfed mitochondria, its complex sample preparation and quantification process can be limiting [17]. Immunoblot assays for mitochondrial protein expression alterations, quantify complete degradation of mitochondrial content, making it difficult to distinguish between mitophagy and degradation by other mechanisms, such as proteasomal degradation pathways and alterations in biogenesis. Similarly, changes in LC3 lipidation, although a gold standard in the autophagy field, cannot differentiate between mitophagy and general autophagy [18]. A combination of western blotting to measure mitochondrial mass and microscopy analysis to colocalize mitochondria with autophagosomes or lysosomes presents an optimal strategy for assessing mitophagy flux. In contrast to other methods, particularly microscopy, flow cytometry offers an excellent means to study heterogeneous cell populations rapidly, minimizing evaluation bias.

Here, we described a new approach for receptor‐mediated mitophagy quantification using flow cytometry as valuable tool for advancing mitophagy research. GFP‐fluorescent‐labeled mitophagy receptor BNIP3L/NIX works as standard outer membrane's mitochondrial marker which enables to monitor exclusively elimination of GFP‐BNIP3L/NIX labeled mitochondrial population after mitophagy induction. Thus, this methodology discriminates between PINK1/Parkin and BNIP3L/NIX eliminated mitochondria. This article presents a detailed protocol for tracking exclusive receptor‐mediated mitophagy using a fluorescently labeled GFP‐BNIP3L/NIX protein. Using any type of fluorescent‐labeled selective autophagy receptor, this method is applicable to the study and quantification of other types of receptor‐mediated selective autophagy.

## Materials

### Cells

HEK293 (ADCC, CRL‐1573).

### Plasmids

pEGFP‐C1/BNIP3L/NIX [14]; pEGFP‐C1/BNIP3L/NIXΔLIR [15]; pEGFP‐C1/BNIP3L/NIXΔTM [13].

### Antibodies and reagents

The following antibodies were used: anti‐GFP (Roche, 11814460001, 1 : 1000, Mannheim, Germany), anti‐p62 (MBL, M162‐3, 1 : 1000, Tokio, Japan), anti‐LC3 (Cell Signaling, 4108S, 1 : 1000, Danvers, MA, USA), anti‐TIMM23 (BD Biosciences, 611222, 1 : 1000, Heidelberg, Germany), anti‐TOMM20 (Santa Cruz Biotechnology, sc‐17764; 1 : 1000, Heidelberg, Germany), and anti‐GAPDH (Sigma‐Aldrich, G9545; 1 : 1000, Saint Louis, MO, USA). Secondary HRP‐conjugated antibodies, goat anti‐mouse (Bio‐Rad, 1706516; 1 : 5000, Hercules, CA, USA), and goat anti‐rabbit (Dako, P0448; 1 : 5000, Santa Clara, CA, USA) IgGs were used for immunoblotting. Donkey goat‐anti‐mouse Alexa Fluor 568 (Thermo Fisher Scientific, A‐11004, 1 : 1000, Waltham, MA, USA) and goat‐anti‐rabbit Alexa Fluor 568 secondary antibody (Thermo Fisher Scientific, A‐11011, 1 : 1000) were used for immunofluorescence. Carbonyl cyanide m‐chlorophenylhydrazone, CCCP (Sigma‐Aldrich, C2759) was applied to cells at a final concentration of 10 μm for 2 or 24 h. 3 μm propidium iodide, PI (Sigma‐Aldrich, P4170) has been used to distinguish dead cells.

## Methods

### Generation of HEK293 cells expressing GFP‐BNIP3L/NIX

HEK293 cells are cultured in complete DMEM supplemented with 5% FBS and 1% penicillin/streptomycin in a humidified cell culture incubator with 5% CO_2_, at 37 °C for 24 h until they reach 60–70% confluency. Upon achieving optimal confluency, cells are transfected with GFP‐BNIP3L/NIX or GFP‐BNIP3L/NIXΔLIR using *jetPRIME* according to instructions. A GFP‐negative sample distinguishes between GFP‐negative and GFP‐positive cell populations. The cells are incubated for 24 h in a humidified cell culture incubator with 5% CO_2_ at 37 °C.

### Mitophagy induction

Transfection efficiency was checked using a fluorescent microscope 24 h after transfection, with an approximate efficiency of 70%. Each transfected cell culture is divided to distribute the cells uniformly according to the number of conditions and treatments planned. The control sample (cells growing in DMSO) should be included. The cells are allowed to attach to the plate surface for at least 6 h to prevent the loss of detached cells after removing the growth medium before the next step. The media is replaced with fresh DMEM containing 5% FBS and 1% penicillin/streptomycin, along with 10 μm CCCP. The cells are incubated for another 24 h in a humidified cell culture incubator with 5% CO_2_ at 37 °C.

**Fig. 1:**
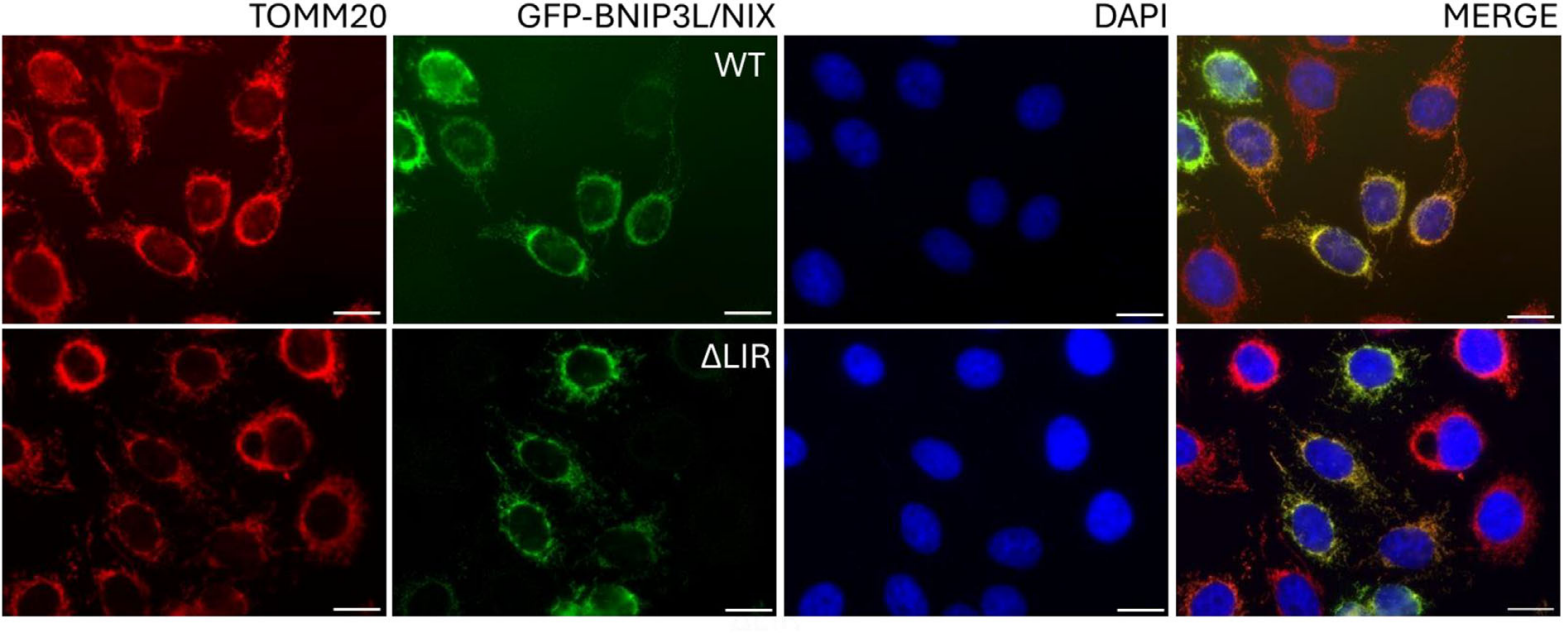
Immunofluorescence detection of BNIP3L/NIX WT and BNIP3L/NIX ΔLIR proteins colocalizing to the mitochondria. The first panel represents mitochondria stained with endogenous TOMM20 (red), the second panel is GFP‐BNIP3L/NIX WT or ΔLIR (green), the third is nuclei stained with DAPI (blue). BNIP3L/NIX and TOMM20 colocalization is reflected as a yellow color in the last panel. Bar = 10 μm.

To ensure that one can apply this method for monitoring receptor‐mediated mitophagy, it needs to be confirmed that receptor indeed localizes to its organelle. In this case, the localization of all tested mutants of the BNIP3L/NIX protein (WT and LIR) to the outer mitochondrial membrane was tested. Here, immunofluorescence microscopy was used as a method to confirm colocalization of the protein with the outer mitochondrial membrane marker, TOMM20 protein (Fig. 1).

### Sample preparation

**Fig. 2:**
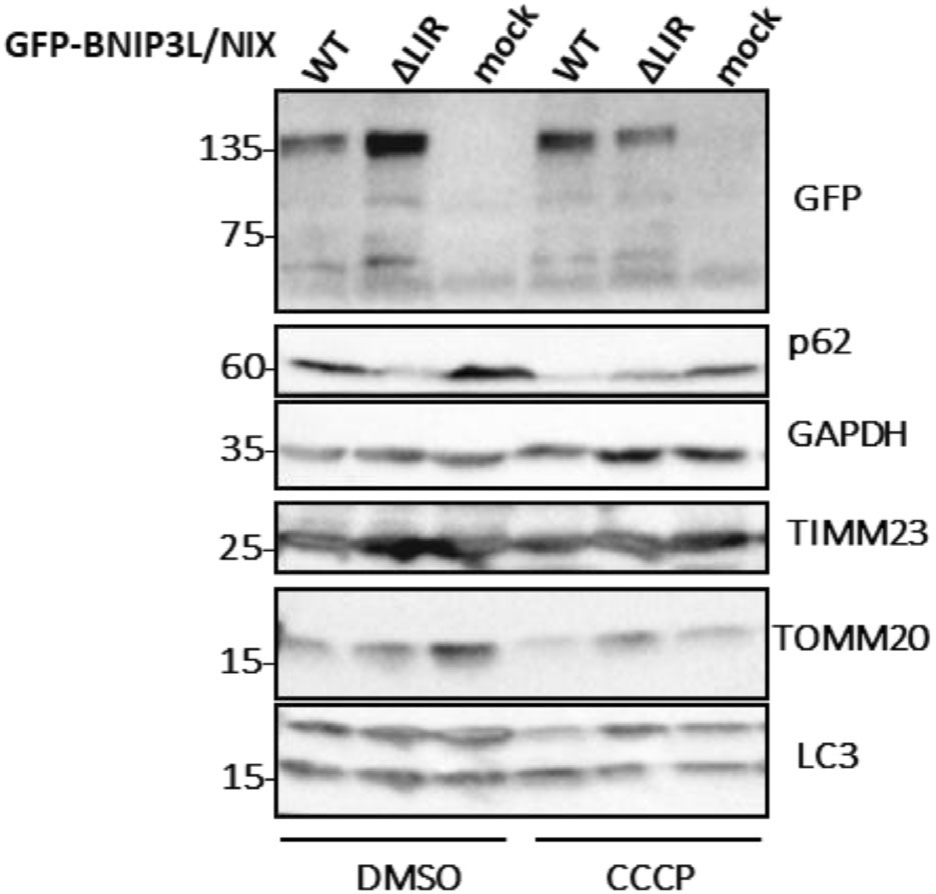
Western blot analysis of protein extracts from HEK293 cells used in flow cytometry. GFP‐BNIP3L/NIX WT and GFP‐BNIP3L/NIX ΔLIR protein expression is confirmed using anti‐GFP. Detection of autophagy is approved using anti‐p62, anti‐LC3 antibodies. Mitochondrial clearance is detected by the presence of mitochondrial markers anti‐TIMM23 and anti‐TOMM20. Anti‐GAPDH is applied as a loading control.

**Fig. 3:**
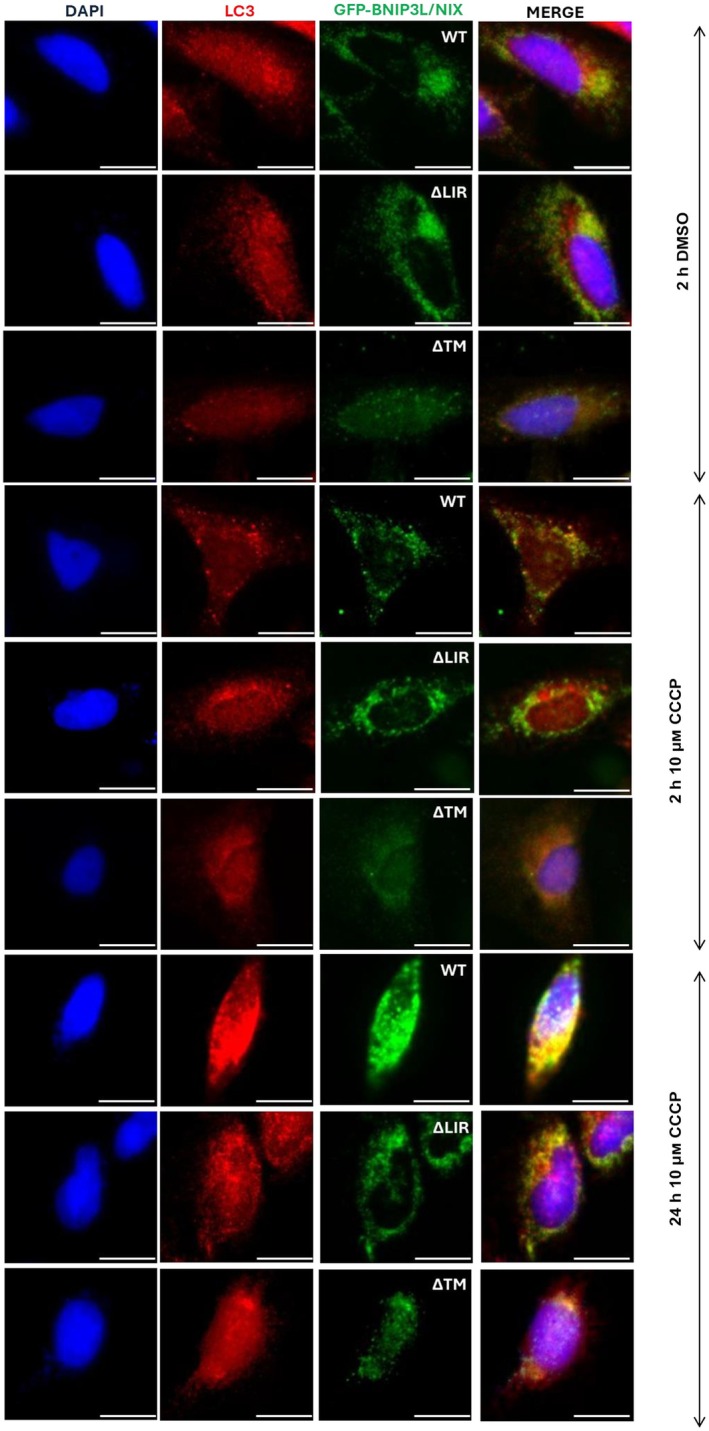
Immunofluorescence detection of LC3 recruitment to mitochondria in cells overexpressing GFP‐BNIP3L/NIX (WT, ∆LIR, and ∆TM) variants. Nuclei are stained with DAPI (the first column), punctate signals represent endogenous LC3A/B protein (red), and the third column signals indicate overexpressed GFP‐BNIP3L/NIX (WT, ∆LIR, and ∆TM) variants (green). Top panel: control cells treated with DMSO for 24 h; middle panel: cells treated with 10 μm CCCP for 2 h; bottom panel: cells treated with 10 μm CCCP for 24 h. Detection of autophagy is approved using anti‐LC3 antibody.

Following treatment, the growth medium is aspirated, and the cells are washed once with 1x PBS. Subsequently, PBS is removed, and the cells are detached using trypsin/EDTA solution to ensure a single‐cell population and prevent cell clumping. Trypsin is inactivated by adding growth medium, containing 10% FBS, in excess. The cells are pelleted at 500 ***g*** for 5 min at room temperature, and the supernatant is discarded. The cell pellet is washed with 1× PBS and filtered through a 70 μm cell strainer to eliminate any remaining cell clumps. Approximately 10^6^ cells are transferred into new microcentrifuge tubes to assess transfection efficiency and treatment effects using western blot and/or immunofluorescence. GFP expression is checked to evaluate transfection efficiency, while LC3 expression is examined to track mitophagy flux (Figs 2 and 3). The cells are pelleted at 500 ***g*** for 5 min at room temperature, and the supernatant is carefully discarded. The pellet is gently resuspended in 1000 μL of viability stain, containing 3 μm PI diluted in 1× PBS, and incubated for 10 min at room temperature, protected from light. The cells are washed twice with 1× PBS and spin at 500 ***g*** at room temperature for 5 min each time. The final cell pellet is resuspended in 1000 μL 1× PBS and analyzed immediately.

### Flow cytometer analysis of mitophagy

#### Flow cytometer settings

HEK293 samples are analyzed using the bd accuri c6 flow cytometer (Becton Dickinson, BD, Franklin Lakes, NJ, USA). Cells are excited with a blue (488 nm) laser, and emission is detected at 533 ± 30 nm with a FL1 detector to track GFP fluorescence and at 585 ± 40 nm with a FL2 detector to detect PI emission. Calibration beads are applied at the beginning of each flow cytometry session to ensure consistency in voltages, maintaining a tolerance of ±10% across previous replicates.

#### Running samples on the flow cytometer

**Fig. 4:**
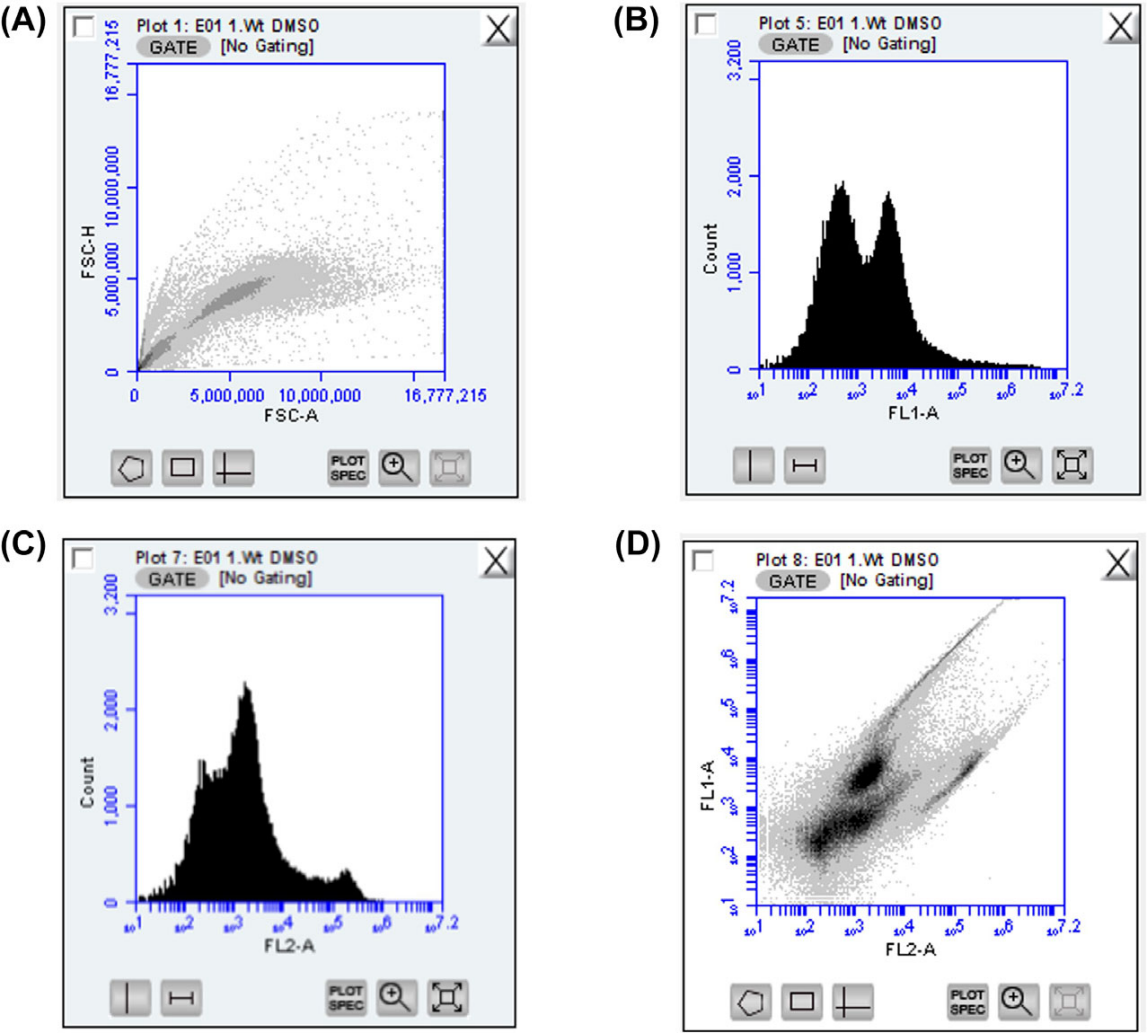
Proposed setup of bd accuri™ c6 software for sample monitoring. (A) FSC‐A versus FSH‐H dot plot for doublet discrimination, (B) FL1‐A versus Count histogram for determination of GFP‐positive cell population, (C) FL2‐A versus Count to monitor the quantity of dead (PI‐positive) cells and (D) Distribution of GFP‐positive and PI‐negative cells in FL2‐A versus FL1‐A dot plot.

For monitoring cell flow, the associated bd accuri c6 software is commonly used. Before running the samples, all necessary parameters for sample collection should be set. When setting events based on a particular population, it is essential to establish the gating scheme on the instrument before data acquisition. However, adjustments to gating can be made during later data analysis if necessary. Typically, a collection limit of 10^5^ events from a 6‐well plate is set. The flow rate should be set to medium (faster rates are generally faster but less precise). To facilitate sample tracking and control, suitable plots and histograms should be configured before running samples (Fig. 4).

#### Data analysis

For data analysis, sophisticated flowlogic™ analysis software is highly recommended. This powerful tool simplifies flow cytometry data analysis with automated features like auto‐compensation and auto‐gating, reducing manual intervention. Its advanced clustering and phenotyping capabilities allow for accurate cell population identification within large datasets. The software provides real‐time data updates, ensuring consistency and immediacy across all linked analysis components. The gating strategy in receptor‐mediated mitophagy analysis is described and illustrated in the following text and scheme.

##### Debris exclusion and doublets discrimination

**Fig. 5:**
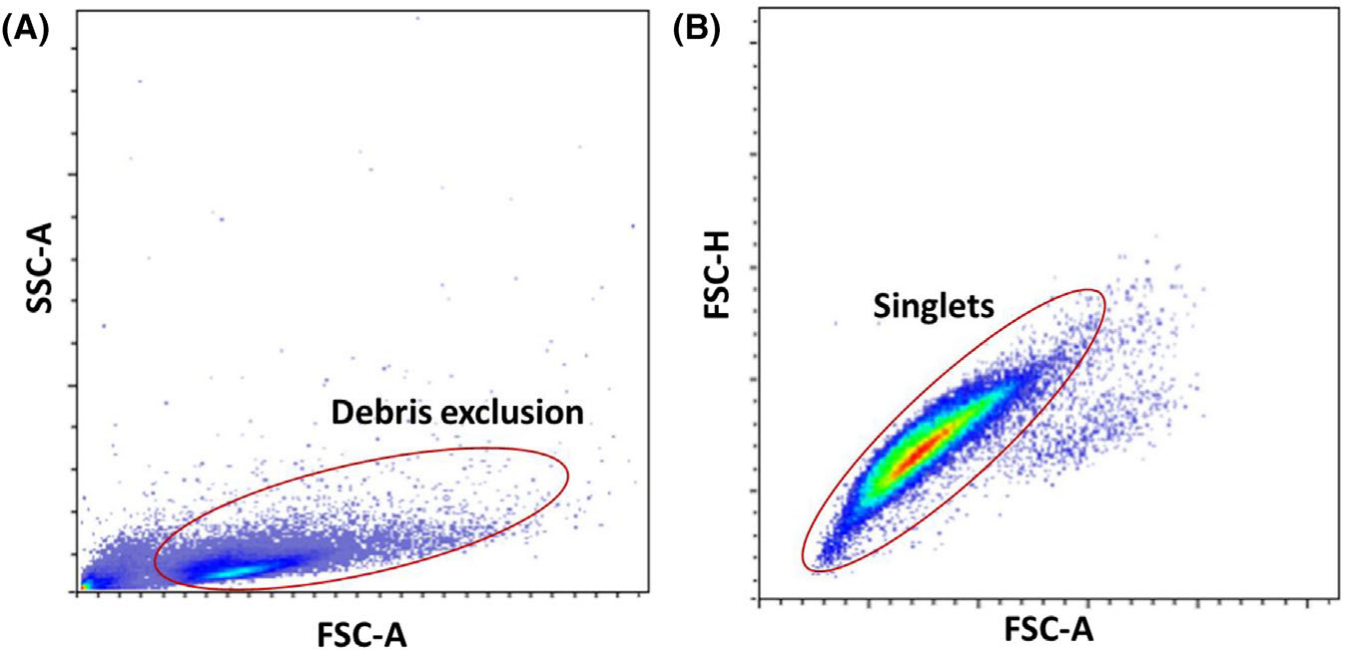
(A) Example of FSC‐A versus SSC‐A plot for debris exclusion. Marked events denote the cell population selected for subsequent analysis. (B) Example of FSC‐A versus FSC‐H plot for doublets discrimination. Gated events represent the cell population selected for following analysis.

Initial gating on FSC‐A versus SSC‐A is used to identify and exclude noncellular debris, ensuring that only intact cells are analyzed (Fig. 5A). After debris exclusion, a bivariate plot with FSC‐H on the *y*‐axis and FSC‐A on the *x*‐axis is created to differentiate single cells (singlets) from cell aggregates (doublets and larger clumps). In this plot, singlets typically form a linear cluster along the diagonal where FSC‐H and FSC‐A values are proportional. A gate is drawn around the linear cluster of singlets, ensuring it is tight enough to exclude doublets and other aggregates but broad enough to include all singlet events (Fig. 5B). This gating strategy is applied consistently across all samples to ensure comparability.

##### Elimination of nonviable cells

**Fig. 6:**
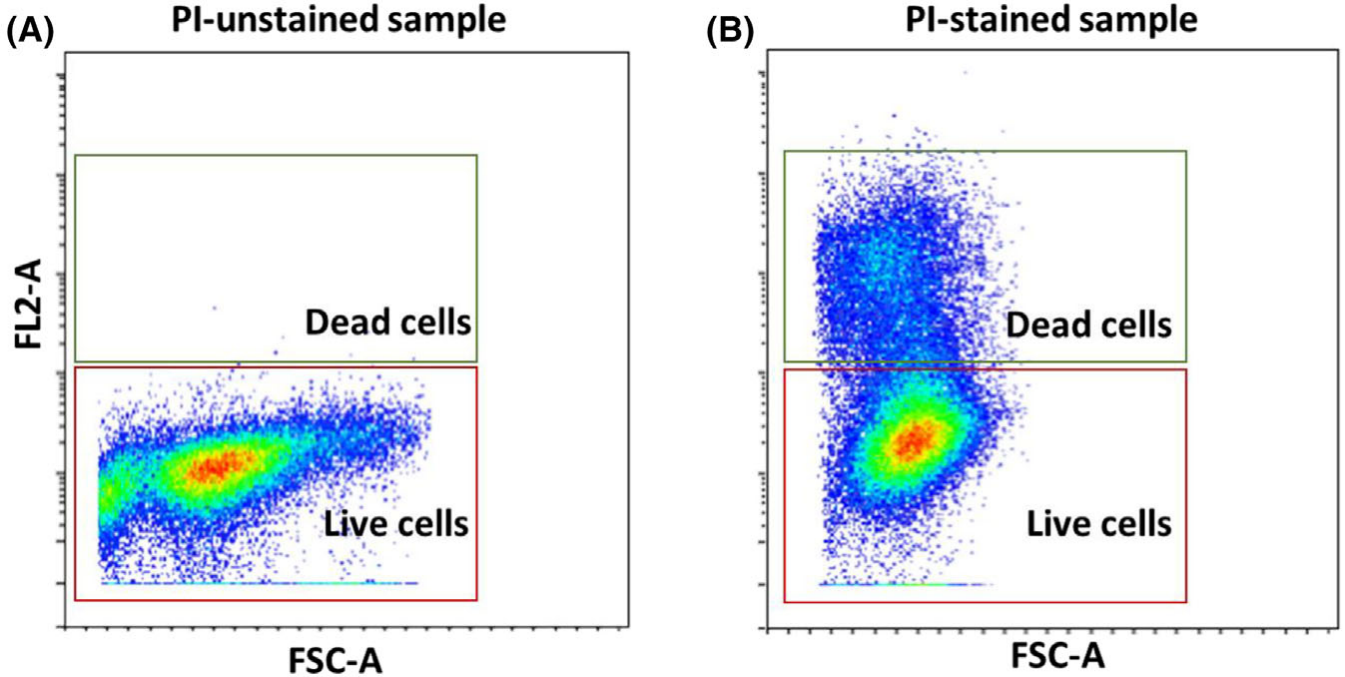
Gating scheme for dead cell elimination. FSC‐A versus FL2‐A dot plot for dead cell exclusion using PI‐unstained (A) and PI‐stained (B) sample. Only live cells are selected for further analysis.

For this analysis, cells are stained with a PI to distinguish between viable and dead cells. Flow cytometry data are collected with FSC and the fluorescence channel for PI (FL2‐A) included. A bivariate plot is created with FSC on the *x*‐axis and FL2‐A on the *y*‐axis. In this plot, two main populations are observed: viable cells, which show low FL2‐A fluorescence due to intact membranes and form a cluster near the *x*‐axis, and nonviable cells (approximately 25% of population), which display high FL2‐A fluorescence due to compromised membranes and form a distinct cluster higher on the *y*‐axis. A gate is drawn around the cluster with low FL2‐A fluorescence, indicating viable cells (Fig. 6).

##### GFP‐positive cell gating

**Fig. 7:**
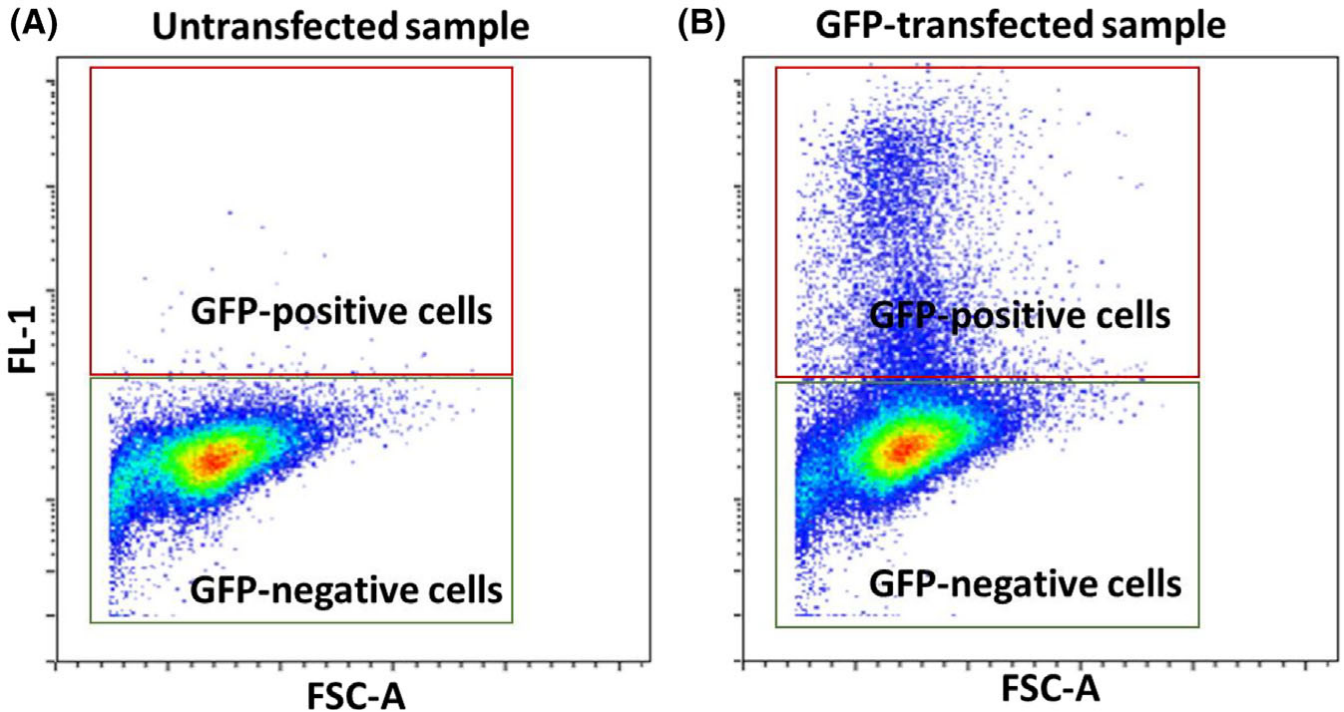
Gating strategy for GFP‐positive cells. (A) An example of FSC‐A versus FL‐1 dot plot for GFP‐negative cell exclusion using an untransfected control. (B) An example of FSC‐A versus FL‐1 dot plot with GFP‐transfected cells. Only GFP‐positive cells are selected for further analysis.

**Fig. 8:**
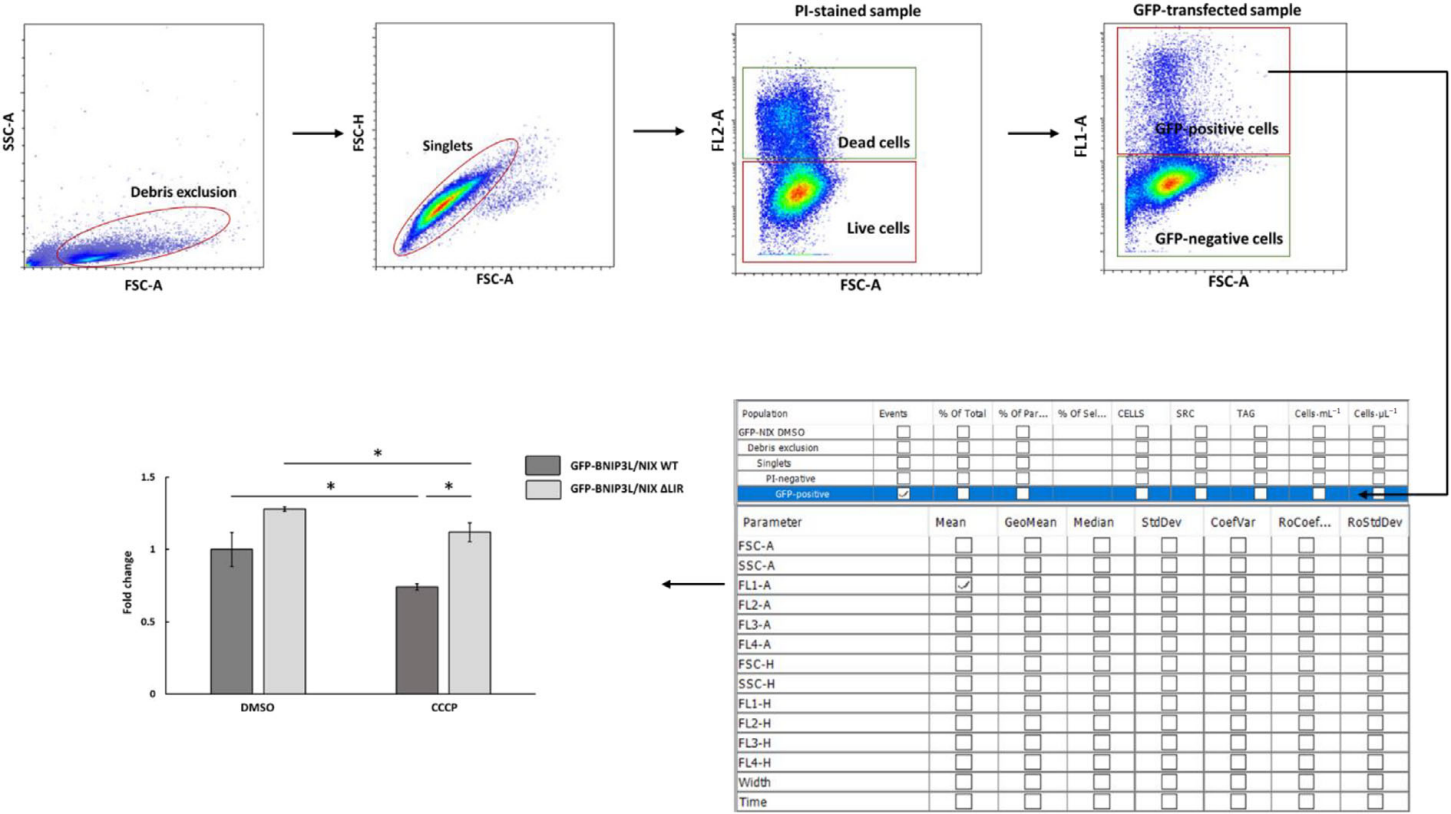
Proposed workflow for detecting mitophagy using fluorescently tagged receptors.

To select and gate for GFP‐positive samples, plots are set up with FSC on the *y*‐axis and the fluorescence channel for GFP (FL1‐A) on the *x*‐axis. A sample containing untransfected cells is used to establish negative gates for GFP fluorescence in the FL1‐A channel (Fig. 7). GFP‐positive events in the FL1‐A channel indicate successfully transfected cells and the mitochondrial population postautophagy induction, since BNIP3L/NIX receptor is exclusively located on the outer mitochondrial membrane and serves as a marker for mitochondria to examine mitophagy progression. To assess mitophagy progression and the number of remaining mitochondria after various treatments, the mean fluorescence intensity in the FL1‐A channel is calculated across at least three independent experiments. The FL1‐A mean should be selected in the statistical parameters, along with the GFP‐positive cell population (Fig. 8). The resulting data are then exported to appropriate data analysis software, such as graphpad prism version 10.0.0 for Windows, graphpad Software, (Boston, MA USA).

##### Statistical analysis and data presenting

**Fig. 9:**
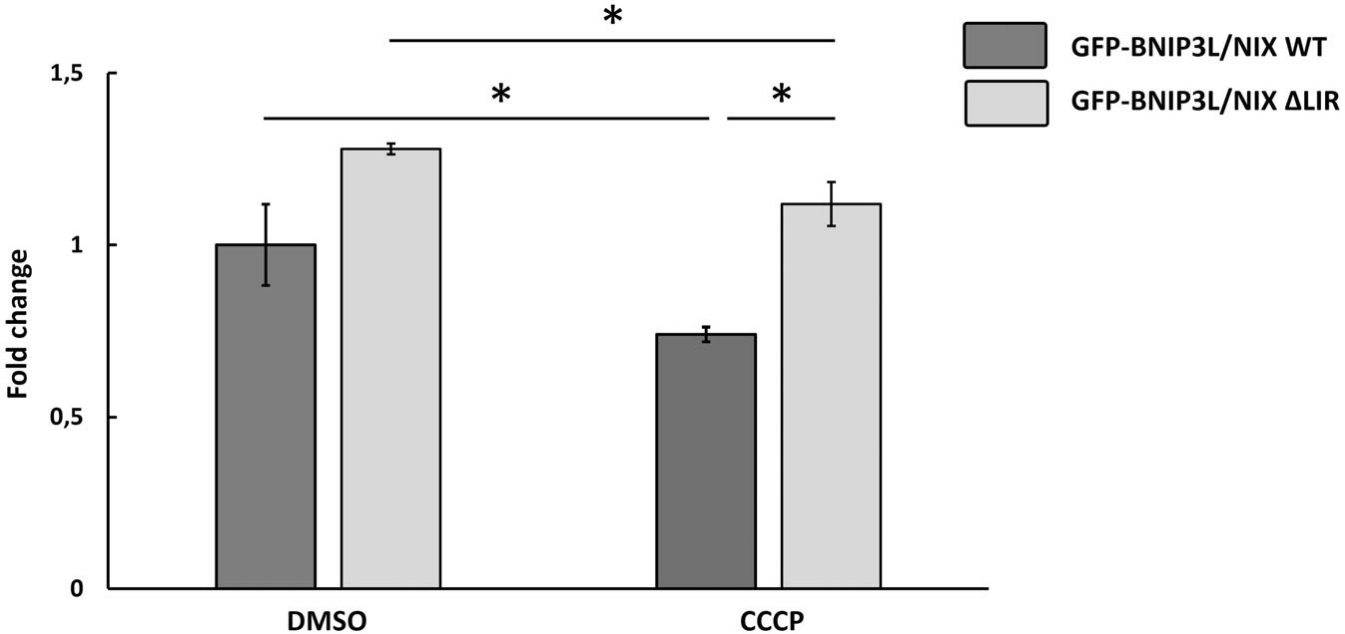
Mitochondrial removal upon CCCP treatment in cells overexpressing GFP‐BNIP3L/NIX WT or GFP‐BNIP3L/NIX ∆LIR proteins monitored by flow cytometry. Transfected cells were treated for 24 h with DMSO or with 10 μm CCCP to induce mitophagy. Two‐way ANOVA with Tukey's multiple comparisons test analysis was used to compare differences between mitochondrial removal in GFP‐BNIP3L/NIX WT and GFP‐BNIP3L/NIX ∆LIR transfected cells. All data were analyzed in graphpad prism 8. Statistical significance: *P < 0.05, error bars indicate standard deviation, *n* = 3.

For final analysis, mean fluorescence intensities in the FL1‐A channel are exported. Statistical analysis is typically performed using graphpad prism 8 software. Here, two‐way ANOVA with Tukey's multiple comparisons test is used to compare differences in mitochondrial removal between cells overexpressing GFP‐BNIP3L/NIX WT and GFP‐BNIP3L/NIX ∆LIR, treated with DMSO and CCCP (Fig. 9).

## Tips and Tricks/troubleshooting

### Generation of HEK293 cells expressing GFP‐BNIP3L/NIX

Ensuring high cell viability is a prerequisite for establishing an optimal experimental setup. Dead cells may nonspecifically bind labeling reagents, leading to elevated background signals and false‐positive results. We recommend the use of HEK293 cells due to several advantages for flow cytometry applications. These cells are easy to handle, exhibit rapid division, and demonstrate high transfection efficiency across various transfection methodologies. One notable advantage of HEK293 cells is their simple and gentle trypsinization process which facilitates rapid detachment from the surface and allows for nonaggressive assessment of single cells.

For transfection, we commonly use *jetPRIME* transfection kit (Polyplus). *jetPRIME* is a robust and versatile DNA transfection reagent suitable for routine experiments, ensuring high efficiency in DNA transfection across various cell types. Its gentle formulation requires minimal amounts of reagent and nucleic acid during transfection, minimizing potential cell damage.

### Mitophagy induction

We recommend preparing a joint transfection for all conditions to maximize synchronization and uniformity of the cell culture, particularly regarding transfection efficiency. This approach can also reduce the initial amount of plasmid DNA used in the experiment.

Our protocol is versatile and can be applied to a wide range of compounds that induce or inhibit autophagy/mitophagy at different levels. Our experience includes the successful use of electron transport chain inhibitors such as Rapamycin, Antimycin A/Oligomycin, or CoCl_2_, which induce mitophagy through hypoxia mechanisms [19] as well as Bafilomycin A1 to block lysosome–autophagosome fusion.

As an additional method to confirm the localization of the autophagy/mitophagy receptors, we also suggest using cell fractionation to verify the presence of the receptor in the specific cell fraction, as we described for BNIP3L/NIX [13].

### Sample preparation

For optimal sample preparation, it is crucial to handle cells with care to minimize potential damage. Additionally, reduce the time from sample collection to processing to maintain cell viability and integrity. Use low vortexing speeds to avoid cell damage and clumping. If possible, use polypropylene over polystyrene tubes, as they reduce cell adhesion and clumping. Our protocol recommends trypsin/EDTA for detaching cells to prevent clumping. However, gentler enzyme solutions like Accutase can also be used, as it is less harsh, maintains cell surface proteins, enhances cell viability, and reduces clumping.

During the final step of sample preparation, cells can be fixed for 15 min with 300 μL of 1% paraformaldehyde to facilitate subsequent analysis on the flow cytometer. Higher paraformaldehyde concentrations can affect some tandem fluorochromes and other fluorescent reagents. However, this process requires delicate handling as paraformaldehyde can render the cell pellet almost invisible. Whenever possible, prepare fresh 1% paraformaldehyde, to avoid efficacy loss from freeze–thaw cycles and polymerization. Avoid prolonged exposure, especially overnight, to prevent increased autofluorescence. Following fixation, cells should be washed twice with 1x PBS and pelleted at 500 ***g*** at room temperature for 5 min each time to remove excess fixative. Note that fixation is incompatible with propidium iodide analysis, but alternative viability fluorescent dyes are available from various manufacturers specializing in flow cytometry.

### Flow cytometer analysis of mitophagy

#### Debris exclusion and doublets discrimination

Operating the flow cytometer at lower speeds and pressures helps to minimize the incidence of doublets by reducing the chances of cells passing through the laser beam in close succession. Ensure that the cell concentration in your sample tubes is optimized to promote good separation between individual cells within the flow cells. Passing your sample through a cell strainer before analysis can remove clumps and aggregates, which contribute to the occurrence of doublets. Make sure to properly dissociate cells and remove any debris or clumps during sample preparation. This can be achieved through gentle pipetting or mild enzymatic treatment if necessary. Using buffers that reduce cell stickiness, such as those containing EDTA, can help in reducing cell aggregation and, consequently, doublets.

#### Elimination of nonviable cells

Here are some tips how to minimize cell death during flow cytometry sample preparation. Maintain optimal conditions throughout the handling process, including keeping cells at physiological temperature and pH, and utilizing prewarmed media and buffers to prevent temperature‐induced stress. Minimize mechanical stress by employing gentle pipetting techniques, and avoiding vigorous shaking or vortexing, which can lead to cell membrane damage. Proper buffer use is crucial like PBS with 2–5% FBS to provide necessary nutrients and maintain osmotic balance. Process samples swiftly to reduce exposure to suboptimal conditions, as prolonged durations can heighten cell death rates. Control cell density to maintain an optimal level (e.g., 10^6^ cells·mL^−1^) to ensure adequate nutrient availability and prevent hypoxic conditions.

#### GFP‐positive cell gating

Regarding GFP expression, it is crucial to optimize transfection efficiency and expression of the recombinant protein GFP‐BNIP3L/NIX across different samples. The level of GFP‐BNIP3L/NIX expression depends on various cellular conditions, among other factors, when working with BNIP3L/NIX protein, transfection time should not exceed 24 h, as prolonged transfection can lead to apoptosis in a significant number of cells (BNIP3L/NIX is also a pro‐apoptotic protein). Additionally, we advise using the same initial transfection conditions for all treatments to further standardize the initial level of GFP‐BNIP3L/NIX expression. Based on our experience, the intensity of GFP fluorescence largely depends on the passage number, so it is most optimal to use cells between passages 4 and 12 and ensure that cells are 60–70% confluent at the moment of transfection.

## Discussion

Mitophagy acts as a protective mechanism by eliminating excess or damaged mitochondria to maintain optimal number of mitochondria in order to balance intracellular homeostasis. Increasing evidence highlights the role of mitophagy as a critical stress response mechanism, essential for cell survival. Therefore, cells have developed various pathways to ensure timely activation of mitophagy under different conditions. Hence, the introduction of novel methodologies for studying mitophagy is crucial for advancing our understanding of this process, its implications for cellular health and disease and for advancing treatment strategies and developing new therapeutic targets.

Here, we present a novel and straightforward method for monitoring BNIP3L/NIX‐mediated mitophagy, which can easily be adapted to detect any other form of receptor‐mediated selective autophagy, including ER‐phagy, xenophagy, and others. In contrast to other available methods for detecting and tracking mitophagy, this method can discriminate between two described types of mitophagy: PINK1/Parkin and receptor‐mediated mitophagy. Furthermore, this method allows for the precise identification of specific receptors involved in mitophagy, knowing the variety of receptors that are unique to different tissues. This specificity provides researchers a powerful tool to explore this crucial process.

The absence of robust and powerful methodologies remains a major barrier in the field of mitophagy. Current methodologies for assessing mitophagy are indeed valuable, however, these techniques come with significant limitations such as difficulties in quantification, potential research biases, and restrictions to *in vitro* studies [16]. Electron microscopy allows for direct visualization of ultrastructural details, including autophagosome‐engulfed mitochondria, providing high‐resolution images that can elucidate the process of mitophagy at a cellular level, but its complex sample preparation and quantification process can potentially limit throughput in large‐scale studies. Also, high‐resolution electron microscopy equipment and expertise are costly and may not be readily accessible to all researchers, limiting widespread use, and accessibility. Immunofluorescence microscopy, a common technique in mitophagy studies, presents several weaknesses that researchers must consider. Similar to other visual techniques, subjective interpretation of fluorescence signals is a significant challenge in immunofluorescence microscopy. Moreover, the technique's resolution may not always be sufficient to distinguish small structures like autophagosomes. Furthermore, the precise quantification of mitophagy events across a large number of cells proves challenging with immunofluorescence microscopy, hindering robust statistical analysis and the reliability of findings. Immunoblot assays may not effectively distinguish between mitophagy‐specific degradation and other cellular processes such as proteasomal degradation or changes in mitochondrial biogenesis. Similarly, LC3 lipidation, while indicating the formation and maturation of autophagosomes, does not differentiate between different types of selective autophagy, such as mitophagy versus general autophagy. This process serves as an early indicator of autophagy initiation but does not directly measure the rate at which cellular components are degraded through the autophagy pathway. To overcome these challenges, researchers often complement LC3 lipidation analysis with additional techniques. Traditional mitophagy bioprobes, such as mt‐Keima, have provided valuable insights by enabling general detection of mitochondrial degradation within cells. However, these tools are limited by their inability to differentiate between distinct mitophagy pathways. Our technology addresses this limitation by utilizing fluorescently labeled mitophagy receptors designed to track specific types of receptor‐mediated mitophagy. This approach offers pathway‐specific insights, allowing for precise monitoring of receptor‐driven mitophagy processes and thereby advancing our understanding of the roles these distinct pathways play.

All of these factors underscore the need for complementary or alternative methodologies to achieve more precise and objective insights into mitophagy dynamics. Flow cytometry offers several advantages for studying and monitoring mitophagy overcoming challenges faced by aforementioned methods. This robust approach enables simultaneous, rapid, and reproducible analysis of a large number of cells, making it essential for studying complex processes like mitophagy. Moreover, flow cytometry provides quantitative measurement of mitophagy levels, reducing subjectivity in data interpretation.

Here, we gave the example of BNIP3L/NIX‐mediated mitophagy monitoring using a previously published BNIP3L/NIX mutant, ΔLIR (LC3 interaction region) [2]. Autophagy receptors bind to LC3 protein through this linear consensus sequence W/YxxL/I (for detailed review refer to [20]). The lack of an LIR domain led to the ablation of BNIP3L/NIX:LC3 binding as well as disruption of LC3 recruitment in cells overexpressing LIR mutant [2]. As an additional approach to prove immunofluorescence evidence of BNIP3L/NIX‐mediated mitophagy activity we have used fluorescence‐tagged BNIP3L/NIX flow cytometry in two of our previous studies [13, 14]. In the first study, Rogov *et al*. demonstrated that mitophagy progression is significantly slowed in cells overexpressing BNIP3L/NIX protein lacking LIR domain compared to cells expressing the wild‐type receptor. Using identical flow cytometry methodology with fluorescently labeled GFP‐BNIP3L/NIX receptor, we proved BNIP3L/NIX receptor dimerization is a crucial mechanism for enhanced activation and progression of mitophagy and further demonstrated that the endogenous protein did not alter the overall findings. [13]. Both studies have demonstrated that this flow cytometry approach involving fluorescently labeled mitophagy receptor, GFP‐BNIP3L/NIX protein, is exceptionally useful and practical for monitoring mitophagy progression compared to immunofluorescence. This approach allows for simultaneous detection of a significantly higher number of cells, and more importantly, this methodological approach, definitely avoid researcher bias in the analysis.

## Conflict of interest

The authors declare no conflict of interest.

## Author contributions

MM conceived and designed the project, acquired, analyzed, and interpreted the data, and wrote the paper; AR acquired the data and interpreted the data; DP analyzed and interpreted the data; IN conceived and designed the project.

## Data Availability

Data sharing is not applicable to this article as no new data were created or analyzed in this study.
